# Synthesis of Core–Shell Micro/Nanoparticles and Their Tribological Application: A Review

**DOI:** 10.3390/ma13204590

**Published:** 2020-10-15

**Authors:** Hao Chen, Lin Zhang, Mengyu Li, Guoxin Xie

**Affiliations:** State Key Laboratory of Tribology, Department of Mechanical Engineering, Tsinghua University, Beijing 100084, China; hao-chen19@mails.tsinghua.edu.cn (H.C.); my-li19@mails.tsinghua.edu.cn (M.L.)

**Keywords:** core–shell micro/nanoparticles, synthesis methods, tribological applications, functionary mechanisms

## Abstract

Owing to the diverse composition, adjustable performance, and synergistic effect among components, core–shell micro/nanoparticles have been widely applied in the field of tribology in recent years. The strong combination with the matrix and the good dispersion of reinforcing fillers in the composites could be achieved through the design of core–shell structural particles based on the reinforcing fillers. In addition, the performance of chemical mechanical polishing could be improved by optimizing the shell material coated on the abrasive surface. The physical and chemical state of the core–shell micro/nanoparticles played important effects on the friction and wear properties of materials. In this paper, the synthesis methods, the tribological applications (acted as solid/liquid lubricant additive, chemical mechanical polishing abrasives and basic units of lubricant matrix), and the functionary mechanisms of core–shell micro/nanoparticles were systematically reviewed, and the future development of core–shell micro/nanoparticles in tribology was also prospected.

## 1. Introduction

In addition to the advantages of both core and shell materials, the core–shell micro/nanoparticles also exhibit special properties far more different from those of simple blends or copolymers due to the formation of core shell structures and the synergistic effect among components [1,2]. Owing to their characteristics of diverse composition, various morphologies, controllable particle size, and synergistic effect among components, the core–shell micro/nanoparticles have been widely applied for adjusting or enhancing the performance of materials, such as optical and electronic properties, bonding property of the interface, catalytic activity, and magnetic thermal induction efficiency [3,4,5,6,7,8,9,10]. Representatively, core–shell micro/nanoparticles have played an important role in the tribology field by acting as a solid/liquid lubricant additive, chemical mechanical polishing abrasives, and basic units of the lubricant matrix.

As for many polymer materials, the molecular chains are connected by a Van der Waals’ force or hydrogen bond, resulting in a relative weak interaction that easily produces relative motion under external force [11]. These properties of polymer materials make them ideal materials for friction reduction and anti-wear because they are prone to form smooth surfaces with very low friction coefficients. Nevertheless, it is difficult for polymers to generate surface adsorption with the traditional solid lubricant filler due to the low surface energy and surface tension, and poor adhesion could result in the reduction of mechanical strength of the polymer composites after the addition of some lubricant particles [12,13]. The core–shell micro/nanoparticles can improve the mechanical properties of the material as a whole, so as to avoid the disadvantages mentioned above while improving the lubricity of the materials because of their good dispersibility and stable interfacial bonding property. Additionally, the core–shell micro/nanoparticles detached during the friction process could easily combine with the friction pair to form a compact transfer film and accumulate on the worn surface of the composites to form a complete lubrication film, which makes the friction actually take place between lubricating films and greatly improves the tribological properties of the materials. Wang et al. [14] prepared SiO_2_@trimethylsilane core–shell nanoparticles using hydrophobic silica nanoparticles and methyl phenyl silicone resin, and they deposited the core–shell particles on poly(ethylene terephthalate) (PET) substrate to prepare a coating with good wear resistance and mechanical durability through plasma treatment and gas phase deposition technology. Another traditional way of friction reduction and anti-wear is to add liquid lubricants with solid additives or directly add solid lubricants to the friction pair. Generally, the additive micro/nanoparticles with high surface energy will continuously aggregate during the relative movement of friction pairs, which will generate large-size clusters and cause damages to friction pairs, thus reducing the anti-wear performance of materials. The core–shell micro/nanoparticles could improve the dispersion of particles by adjusting and optimizing the shell materials and controlling the physical and chemical state of the surfaces, so as to achieve low friction and good service reliability. Lubricant-dispersible nanoparticles with surface modification were synthesized, and the tribological properties of the nanoparticles as lubricant additives showed that the addition of nanoparticles reduced wear and increased load-carrying capacity remarkably, which indicated that core–shell micro/nanoparticles can be applied as anti-wear additives with excellent performance [15]. As for chemical mechanical polishing, traditional abrasive particles such as SiO_2_ and CeO_2_ with irregular shape and hard texture will easily scratch the polished surface, thus reducing polishing quality. The core–shell micro/nanoparticles are generally spherical and can play the role of ball bearings to reduce mechanical damage during polishing process. Meanwhile, the soft core materials can perform elastic deformation, resulting in the increasing of the actual contact area between the abrasive and the matrix and reducing the contact stress and effectively preventing scratches on the matrix. What’s more, the hard shell materials ensure that the abrasives will not be too soft and cause insufficient grinding. A novel organic/inorganic core–shell structured abrasive PS@SiO_2_ with smooth surface and controllable size was presented, and the slurry containing PS@SiO_2_ abrasives exhibited much better surface quality than that containing SiO_2_ abrasives (root mean square (RMS) surface roughness reduced from 4.27 to 0.56 nm) [16].

It has been an important research direction in the tribology field to explore high-performance lubricating materials with low friction and high wear resistance and stable, efficient polishing abrasives. The design idea of core–shell micro/nanoparticles is a chemical or physical combination of hard and soft materials at the microscopic level, so as to maintain the properties of core materials and enhance their dispersibility and environmental tolerance. It is worth mentioning that the structure, size, and morphology of core–shell particles have different effects on the friction and wear properties of materials. Therefore, this paper summarizes the preparation methods, lubrication and wear resistance properties, and mechanism of core–shell particles in the hope of providing reference for the design and development of new solid lubricating materials, liquid lubricating material additives, and polishing abrasives.

## 2. Synthesis of Core–Shell Micro/Nanoparticles

In general, the core–shell micro/nanoparticles are fabricated in two steps: the preparation/synthesis of core materials and synthesis/deposition of shell layers. According to the different principles, the synthesis methods of nanoparticles can be divided into four categories: solid phase reaction, liquid phased reaction, gas phase reaction, and mechanical mixing.

### 2.1. Solid Phase Reaction

The process of the solid phase reaction method is that the core materials and shell precursors are sufficiently mixed and calcined according to a formula to obtain superfine coated particles. There are ways to achieve the goals of mixing, including solution dispersion, decomposition, mechanical stirring, grinding, and ball-milling. The solid phase reaction method is usually used to synthesize the materials that need phase transformation to gain specific properties by calcination under high temperature, which cannot be achieved by wet chemistry approaches during the synthesis process. Consequently, this method is usually used for the synthesis of inorganic core–shell nano/microparticles.

**Figure 1:**
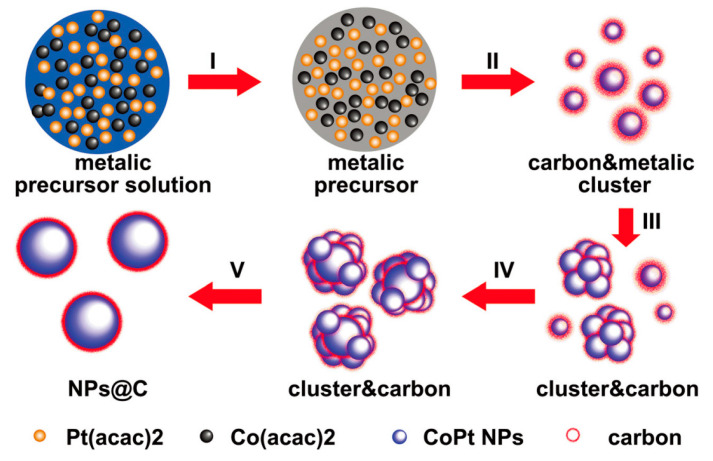
Schematic representation of the formation of L1_0_-CoPt@C nanostructures [17]. Reproduced with permission from [Baoru Bian et al.], [Nanoscale]; published by [Royal Society of Chemistry], 2015.

An one-step solid phase synthesis method was reported to synthesize monodisperse single crystal Co(Fe)Pt@C nanoparticles with an L1_0_ structure; as shown in Figure 1, the metal salts were dispersed, dried, and decomposed on the carbon-coated copper grid, followed by a cluster formation process (I, II, III). Then, the grains in the clusters aggregated and recrystallized to form single crystal core–shell nanoparticles (IV, V) [17]. The synthesized particles exhibited ferromagnetic properties with coercivity up to 12.2 kOe at room temperature, which cannot be attained by a traditional two-step synthesizing method that includes the solution phase synthesizing of fcc (face-centered cubic)-Co(Fe)Pt nanoparticles.

**Figure 2:**
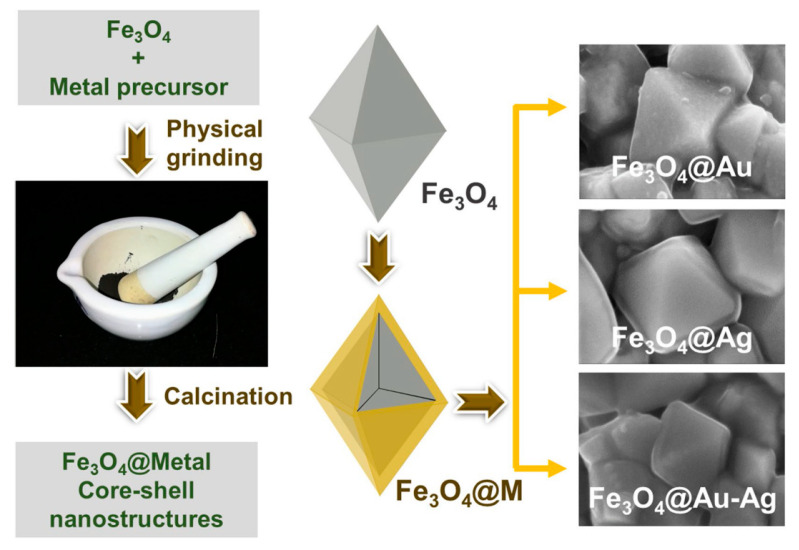
Schematic representation to obtain Fe_3_O_4_@M core–shell nanostructures [18]. Reproduced with permission from [Srinivasa Rao et al.], [Nature]; published by [Springer Nature], 2019.

Catalytically active Fe_3_O_4_@M (M = Au, Ag and Au-Ag alloy) core–shell nanostructures were fabricated through a solid state synthetic approach by Nalluri et al. [18], and it involved a simple physical grinding of metal precursors over Fe_3_O_4_ particles, followed by calcination, which was cost-effective, quantifiable, eco-friendly, and simple when compared with commonly used solution-based methods. The schematic of the synthetic procedure and the morphology of products are shown in Figure 2.

When considering a solid-phase reaction method, the crucial steps are the mixture and calcination of particles. Therefore, some limits should not be ignored. Normally, the raw materials and products are solid and contact and mix with each other in a particle state of a few microns, which will cause insufficient contact between raw materials and lead to inadequate reactions. What is more, in the process of nucleation, the lattice structures and atomic arrangements of the raw materials have to be greatly adjusted or rearranged, which will take a large quantity of energy and can only be achieved at high temperatures because of the great structural differences between the products and the raw materials.

### 2.2. Liquid Phased Reaction

The liquid phase reaction method refers to the chemical reactions under a wet environment to deposit modifiers or film on the surface of pre-formed particles [19]. Compared with other methods, the liquid phase reaction method has the advantages of simple equipment and low reaction temperatures, and it is widely used in the laboratory and industry to prepare core–shell nano/microparticles. The commonly used liquid phase reaction methods include the sol–gel method, hydrolysis method, electrochemical method, hydrothermal method, and emulsion polymerization.

For the sol–gel method, the core materials and shell precursors were dispersed in solutions, which was followed by the formation of active monomers through hydrolysis reactions of precursors. Afterwards, the active monomers polymerize to form sols and then generate gels with certain spatial structures on the surface of core materials. After drying and heat treatments, the desired core–shell particles are prepared. A two-step sol–gel method was developed by Wang et al. [20], and they added ether and H_2_O in sequence to a polysulfone (PSF)-containing dimethyformamide (DMF) solution to facilitate PSF solidifying on the surface of a glass bead to prepare monodispersed glass bead@polysulfone microspheres. Their results proved that the two-step sol–gel method is a highly efficient process for the preparation of glass bead@polysulfone microspheres with 80% and almost 100% utilization rates of PSF and glass beads, respectively. The sol–gel method is the most commonly used way to synthesize SiO_2_ core–shell particles through the hydrolysis of TEOS (tetraethyl orthosilicate). Marini and co-workers [21] managed to prepare organic–inorganic hybrid core–shell nanoparticles with a PE–PEG (α-hydroxy-terminated poly(ethylene)-block-poly(ethylene glycol)) amphiphilic tapered shell and an inorganic-rich core (SiO_2_) in a high yield by a simple one-step catalyzed sol-gel reaction using TEOS and triethoxysilane-terminated polyethylene-b-poly(ethylene glycol) as reactants with low cost. What is more, SiO_2_ nanoparticles with a covalently grafted PEtOx(poly(2-ethyl-2-oxazoline)) shell were fabricated by the sol–gel method [22]. Afterwards, SiO_2_@PEtOx particles were transformed into SiO_2_@P(EtOx-*stat*-EI) under acidic conditions to invert the surface charge from negative to positive, which could be used to deposit Au nanoparticles subsequently for nonviral gene delivery.

In summary, the raw materials used in the sol–gel method need to be dispersed into the solvent to form a low-viscosity solution first. Therefore, it is easy to form gels and achieve doping with great uniformity in molecular levels. However, there are also some problems with the sol–gel method that cannot be ignored. Generally, the whole sol–gel process will take several days or even weeks, and there can be a lot of micropores in the gels, which will lead to the shrinking of samples due to the escaping of gases and organic compounds during the drying process.

**Figure 3:**
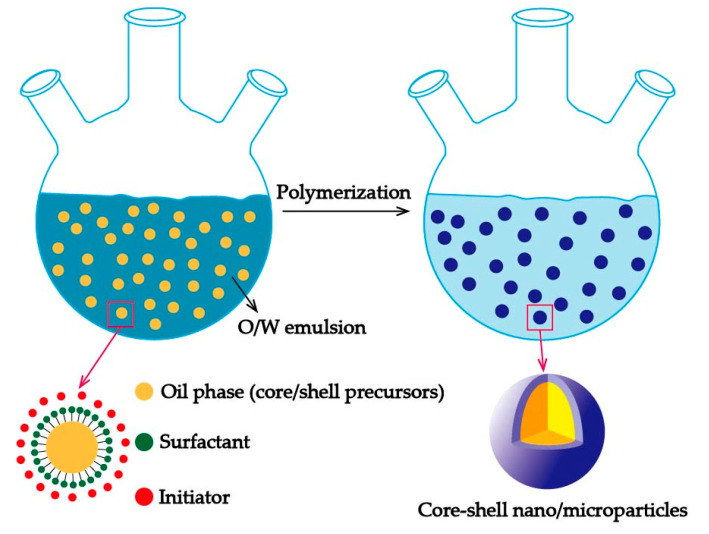
Schematic representation of the oil in water (O/W) emulsion polymerization.

In addition to sol–gel method, emulsion polymerization is another pop method for the synthesis of core–shell micro/nanoparticles. The most common used type of emulsion polymerization is an O/W (oil in water) emulsion, which usually starts in the water containing core materials, monomers, initiators, and surfactants, as shown in Figure 3. During the process, the droplets of oil mixture (core materials and monomers) are emulsified in the water under mechanical stirring or ultrasonic with the help of surfactants. Then, the monomers will polymerize under the action of initiators, and the phase separation will take place within the droplets to obtain core–shell particles. Researchers have reported some improved ways of the emulsion polymerization method to prepare their targeted core–shell particles. Sun and his team [23] fabricated the SiO_2_@polypyrrole composites by the in situ polymerization of pyrrole monomer on the surface of SiO_2_ spheres, which were prepared with the sol–gel method in advance. A seeded surfactant-free emulsion polymerization was demonstrated by Diego et al. [24] to synthesize PTFE (polytetrafluoroethylene)@PMMA (polymethyl methacrylate) core–shell particles with the diameters of 100–350 nm. They managed to polymerize MMA (methyl methacrylate) monomers on the surfaces of PTFE seeds without the surfactant and demonstrate that the method is highly efficient in preparing core–shell nanoparticles featuring monodisperse and narrow size distributions.

The emulsion polymerization can be carried out with fast reaction speed, high molecular weight, and controllable polymerization temperature, and the polymerization system has low viscosity even in the later stage of the reaction. Therefore, the emulsion polymerization method is suitable for the preparation if high-viscosity polymers. In general, the particle size, dispersity, and shell thickness of core–shell particles can be greatly influenced by the stirring rate and the type and content of the surfactants; usually, the high stirring rate can result in the smaller particle size.

For the hydrolysis method, it is similar to the sol–gel method but without the sol–gel process. It refers to the direct chemical depositions of the shell (usually inorganic, such as SiO_2_, TiO_2_, and ZrO_2_) precursors on the surfaces of the prepared core materials such as Au, Ag, ZnO, CdTe, CdSe, and CaCO_3_ through hydrolysis reactions [25,26,27,28,29]. The hydrolysis reaction time can greatly influence the thickness of the shells (the longer the hydrolysis time, the thicker the shell layer), so as to effectively regulate the properties of core–shell particles. Kumar et al. [30] studied the electrooxidation of CO and methanol on Pt thin films deposited onto 3 nm Au particles using the electrochemical method, and they found that the electrooxidation depended strongly on the Pt film thickness and Au nanoparticle coverage. A simple method to prepare carbon-coated Fe_3_O_4_ nanoparticles (Fe_3_O_4_/C) by a hydrothermal reaction was presented [31], and the Fe_3_O_4_/C nanoparticles were applied as solid-phase extraction (SPE) sorbents to extract trace polycyclic aromatic hydrocarbons (PAHs) from environmental water samples for the first time.

Due to the convenience of the liquid phase reaction method mentioned above, this method is the most common way for researchers to obtain the core–shell particles in laboratories. In addition to the advantages, the liquid phase reaction has the characteristics of individual self-nucleated shell particles, lack of scalability, the requirement of further purification, and harmful organic solvents, which can restrict the further development of the liquid phase reaction method and need to be improved in the future.

### 2.3. Gas Phase Reaction

The gas phase reaction method is used to deposit the shell materials in the gas phase supersaturated system on the surfaces of the target particles to form the core–shell nano/microparticles, and the method mainly includes physical vapor deposition (PVD) and chemical vapor deposition (CVD).

The technical principle of PVD is that the material sources are vaporized into gaseous atoms, molecules, or partially ionized into ions by means of physical methods under the vacuum conditions and transported by low-pressure gas or plasma to deposit on the surfaces of the core materials with certain functions. The PVD method mainly includes cathodic arc deposition, electron beam physical vapor deposition, evaporative deposition, pulsed laser deposition, and sputter deposition. Rh-Au core–shell nanoparticles were successfully obtained on the TiO_2_ (110) surface by the PVD of Rh followed by the exposure of Au at an elevated sample temperature (500 K) [32], which proved that the “seeding and growing” method for the growth of monometallic particles in narrow size distribution [33] could also be used in the fabrication of bimetallic nanoparticles. Fe@Au core–shell nanoparticles synthesized by sequential deposition from two elemental targets in an ultrahigh vacuum sputtering device were reported [34], and the particles displayed an original polyhedral morphology and stability of the remarkable centered core–shell morphology, which demonstrated that a complete gold shell could be grown on an iron nanocrystal, thus preventing the core from oxidation and ensuring the biocompatibility of the nanoparticles. PVD coatings are harder and more corrosion resistant, and the core–shell particles fabricated by this method have higher impact strength, temperature, and abrasion resistance. However, specific technologies can impose constraints, some PVD methods must be operated at very high temperature and vacuums, causing a limit to the further applications of the method in both the laboratory and industry.

**Figure 4:**
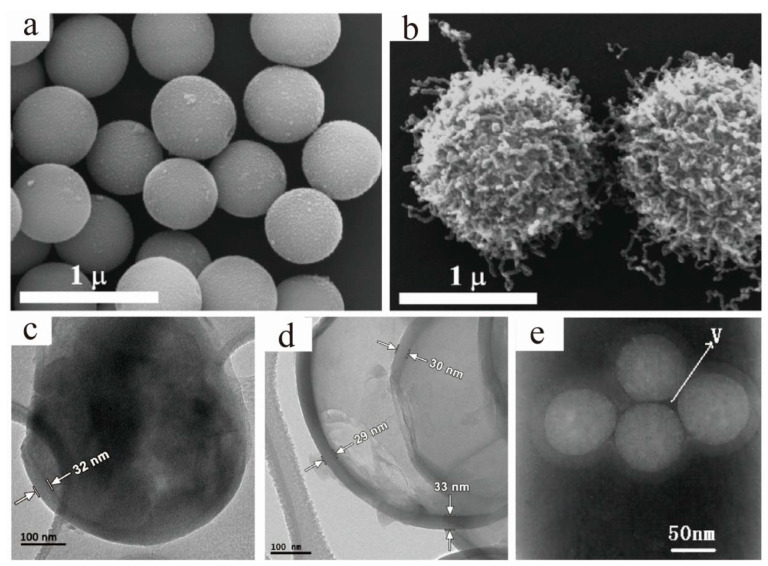
Scanning electron microscope (SEM) images of (**a**) SiO_2_/IO microspheres and (**b**) “Medusa-like” SiO_2_/IO/carbon nanofibers and tubes particles [35]. Reproduced with permission from [On Mero et al.], [Langmuir]; published by [American Chemical Society], 2014; Transmission electron microscope (TEM) images of the 1 µm Li_2_S@C core–shell particles (**c**) before and (**d**) after dissolving Li_2_S [36]. Reproduced with permission from [Caiyun Nan et al.], [Journal of the American Chemical Society]; published by [American Chemical Society], 2014; (**e**) TEM micrographs of four cores over-coating with one shell structure SiC/SiO_2_ nanoparticles [37]. Reproduced with permission from [LianZhen Cao et al.], [Journal of Alloys and Compounds]; published by [Elsevier], 2010.

As for CVD, it mainly includes the chemical reactions of gases or steams on the surfaces of core materials to form target core–shell particles. The CVD method can be divided into the plasma method, atomic-layer CVD (ALCVD), combustion CVD (CCVD), hot filament CVD (HFCVD), hybrid physical–chemical vapor deposition (HPCVD), rapid thermal CVD (RTCVD), and photo-initiated CVD (PICVD). Furthermore, CVD is usually used to deposit materials in ways that traditional techniques are not capable of. Mero et al. [35] fabricated “Medusa-like” SiO_2_/IO (Iron Oxide)/carbon nanofibers and tubes particles via the CVD of ethylene on the surfaces of the SiO_2_/IO microspheres that were previously prepared by the solid phase reaction method, as shown in Figure 4a,b. The particles they synthesized had the potential uses for catalysis, environmental remediation, and various biomedical applications. Through the CVD method, the previously synthesized Li_2_S spheres were converted into stable Li_2_S@C core–shell particles (Figure 4c,d), and the particles showed promising specific capacities and cycling performance with a high initial discharge capacity of 972 mAh g^−1^ at the 0.2C rate [36]. A novel route of thermal CVD and thermal annealing process was presented and used to synthesize SiC@SiO_2_ core–shell nanoparticles with good blue emission (Figure 4e), which indicated that the particles had potential applications in blue-light optoelectronic devices [37]. In contrast to direct evaporation and deposition, the CVD method can produce a variety of thin films (carbides, nitrides, borides, silicides, and oxides) at the deposition temperatures well below the melting points or decomposition temperatures, and the reaction source materials needed for film formation are generally easy to obtain. What is more, the compositions and characteristics of the films can be easily controlled by consciously changing and adjusting the compositions of the reactants. However, the low deposition rate, high equipment requirements, and damages to the environment because of the flammable, explosive, or toxic residual gases should not be ignored.

Both PVD and CVD are widely used for the preparation of thin films and core–shell particles; however, the tribological applications of core–shell particles synthesized by those methods are rarely reported at the present. Therefore, they are worth researchers’ attention.

### 2.4. Mechanical Mixing

**Figure 5:**
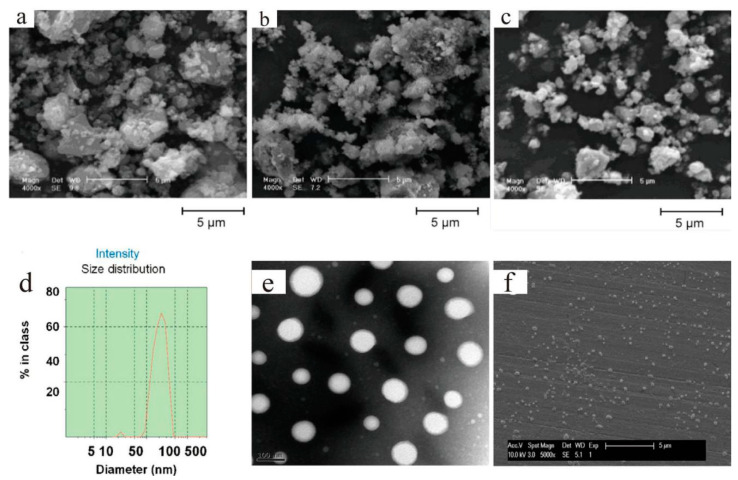
Morphology of powder particles (stoichiometric composition) after (**a**) 90 min, (**b**) 120 min, and (**c**) 240 min of milling times [39] Reproduced with permission from [A. Heidarpour et al.], [Journal of Alloys and Compounds]; published by [Elsevier], 2009; characterization of the gel nanocarriers (NCs). (**d**) Particle size and size distribution, (**e**) TEM and (**f**) SEM images of the gel NCs [40]. Reproduced with permission from [Wei He et al.], [International Journal of Pharmaceutics]; published by [Elsevier], 2015.

The mechanical mixing method means that two or more particles with good adhesion or adsorption properties at a certain temperature can be evenly adsorbed on the surfaces of the modified particles by convection and diffusion. Afterwards, under the action of external forces (aerodynamic force, gravity, and mechanical force), the particles are closely combined to cover the surfaces of the target particles, thus completing the surface modification or recombination [38]. Heidarpour and co-workers [39] synthesized Mo@Al_2_O_3_ nanocomposites by ball milling of Al and MoO powder mixtures to take a rapid combustion reaction process, and the SEM images of the products can be seen in Figure 5a–c. In addition to ball milling, temperature-sensitive and core–shell structured gel nanocarriers (NCs) for paclitaxel (PTX) with 12-hydroxystearic acid as an organic gelator were fabricated by a mechanical mixing and high-pressure homogenization method (Figure 5d–f) [40]. The PTX-loaded gel NCs had better in vivo biocompatibility and could suppress tumor growth more efficiently than traditional nanocarriers without harming other organs.

The mechanical mixing method can improve the properties of coated particles to some extent. However, it is difficult for this method to completely cover the surfaces of particles uniformly due to the weak bonding forces between the core and shell materials; therefore, it is rarely used for the preparations of core–shell micro/nanoparticles at present.

## 3. Applications

The tribological applications of core–shell nano/microparticles mainly focus on the fillers in a solid lubricant matrix, additives in liquid lubricants, and composite abrasives in chemical mechanical polishing to achieve a low COF (coefficient of friction), high wear resistance, and stable, efficient polishing properties with the formation of core shell structures and the synergistic effect among components [1,2]. It is worth mentioning that some core–shell particles are directly used as basic units of the bulk composites in recent works.

### 3.1. Fillers in Solid Lubricant Matrix

The addition of nano/microparticles into solid materials can improve both the tribological and mechanical properties, which can be obviously influenced by the structures and dispersions of nano/microparticles. The traditional mechanical mixing of fillers and matrix has the problem of phase separation or uneven dispersion of the particles, resulting in the decreases of mechanical properties of the composites, which cannot meet the technical requirements of low friction, high wear resistance, and mechanical properties of solid lubricating materials under harsh working conditions. Compared with ordinary particles, core–shell nano/microparticles possess the shell materials that can interlock the matrix and particles as well as improve the dispersion of particles in the matrix. Meanwhile, the core materials can play the roles of wear resistance, friction reduction, and fortifier. Typically, the incorporation of rigid nano/microparticles into the polymer matrix could improve the mechanical properties of the composites due to the tolerance of the particles to large plastic deformation under external forces [41,42,43]. Consequently, core–shell nano/microparticles have a broad application prospect as fillers in a solid lubricant matrix.

#### 3.1.1. Enhanced Mechanical Properties

**Figure 6:**
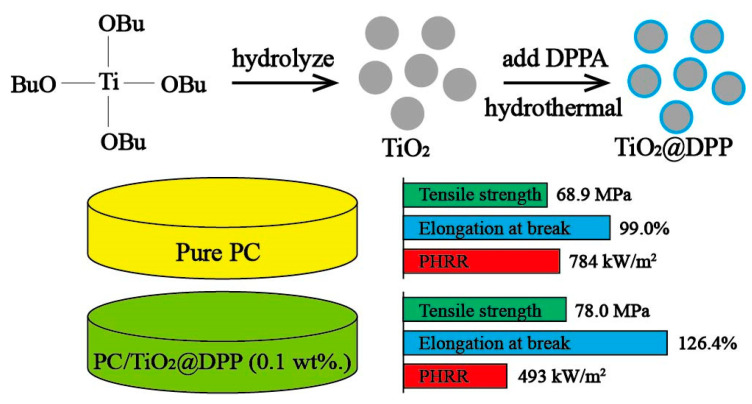
Schematic representation of the formation of TiO_2_@DPP and the enhancement of the composites [43]. Reproduced with permission from [Yunxia Wei et al.], [Applied Materials]; published by [American Chemical Society], 2018.

In general, the mechanical enhancements of the composites are attributed to the properties of core materials such as rigidity or the ability that can provide effective load transfer in the composites system [41,42,43] and the interlock between shell materials and matrix. In this paper, the parameters of the typical core–shell nano/microparticles strengthening mechanical properties of the matrix reported so far are summarized in Table 1 and Figure 6 and described in detail.

**Figure 7:**
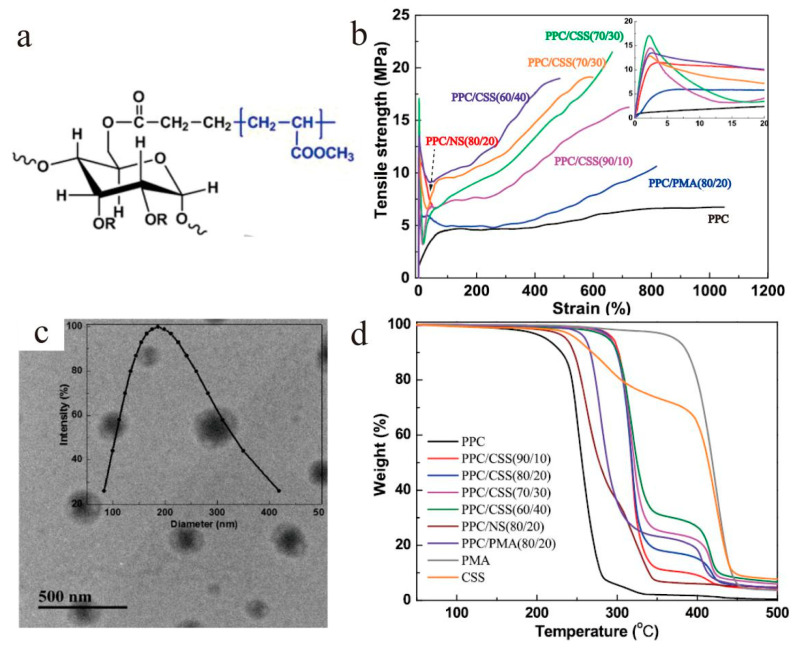
(**a**) Molecular structure of PMA grafted starch; (**b**) stress−strain curves of PPC, PPC/core–shell starch (CSS), PPC/native starch (NS), and PPC/PMA (propylene glycol methyl ether acetate) composites; (**c**) TEM image of core–shell starch nanoparticles (CSS NPs); (**d**) thermo gravimetric analysis (TGA) curves of samples [50]. Reproduced with permission from [Li Liu et al.], [ACS Sustainable Chemistry & Engineering]; published by [American Chemical Society], 2019.

As mentioned above, the mechanical properties of composites can be greatly influenced by the fillers’ properties and dispersion in matrix. In general, inorganic components in the composites contribute better mechanical, thermal and anti-wear performances, but they tend to phase separate and aggregate due to the size effect. To solve these problems, researchers have presented different means. TiO_2_ particles were coated with diphenylphosphinic (DPP) to improve the dispersion properties of TiO_2_ particles in the polycarbonate (PC) matrix [43]; the SEM results illustrated the uniform distribution of the TiO_2_@DPP in PC. Moreover, the TEM images showed indistinct interfaces between the TiO_2_@DPP particles and PC, which meant good interface interaction between the materials. The composite (0.10 wt % TiO_2_@DPP) possessed great fire retardance and better mechanical properties, as shown in Figure 7. Mekuria et al. [44] developed SiC@SiO_2_ by the thermal oxidation of SiC nanoparticles, and the SiC@SiO_2_ particles were amino-functionalized by APTMS (3-aminopropyltrimethoxysilane) to achieve the uniform dispersion of the nanoparticles in the PI matrix and prevent stacking and aggregating. As a result, the tensile strength and Young’s modulus were significantly improved compared to the neat PI (77.1% and 96.6% higher, respectively). To improve the dispersion of SiO_2_ in the polyvinyl chloride (PVC) matrix and the surface interaction between SiO_2_ and PVC, PMMA was covered on SiO_2_ by emulsion polymerization to prepare SiO_2_@PMMA core–shell nanoparticles [45]. The SiO_2_@PMMA/PVC composites exhibited great mechanical enhancements (both tensile strength and elongation at break were increased about 100%). Chatterjee and co-workers [47,48] encapsulated nano-CaCO_3_ in different shell materials (PS and PMMA) to achieve perfect dispersion and mechanical enhancement in different matrixes (PP and PS), as shown in Table 1.

In addition to inorganic core materials, core–shell nano/microparticles with organic cores are also of great importance in improving the mechanical properties of a matrix [50,51,52,53]. Starch@PMA nanoparticles were fabricated and induced a significant increase in the mechanical properties of poly(propylene carbonate) (PCC) (the tensile strength increased by 3.2-fold compared to neat PPC) due to the rigidity of the starch hard core as shown in Figure 7 [50]. To increase the interfacial adhesion of natural rubber (NR) to the PMMA matrix, the NR particles were coated with a rigid poly(methyl methacrylate)-co-poly(3-trimethoxy silylpropyl methacrylate) (PMMA-co-PMPS) shell, which could give good compatibility with PMMA and increase the mechanical properties of the composites [51]. Dispersing elastomeric core–shell structured materials into brittle and notch-sensitive polymers is a common way to improve the impact strength. The cores of the materials can absorb the impact, while the shells can increase the compatibility with the matrix. Misuk et al. [52] developed butyl acrylate rubber (BAR)@MMA particles as elastomeric impact modifiers in a CDA matrix with good interfacial interaction and impact strength. Impressively, the impact strength of PBT (polybutylece terephthalate) composites was successfully improved by 8.64-fold by introducing poly(n-butyl acrylate)@PMMA core–shell particles into neat PBT [53]. It is a very promising approach to achieve well-compatibilized PBT composites with high toughness.

For mechanical enhancements of a solid matrix by nano/microparticles, the key is to increase the dispersion of the particles in the matrix and the interfacial interaction between the particles and the matrix. Coating the core materials with shells that are compatible with the matrix or surface modifying the particles with certain functional groups are ideal methods to achieve the goals. Meanwhile, the mechanical properties of core materials can greatly affect the mechanical performance of the composites; in general, inorganic cores can improve the tensile strength, thermal, and anti-wear properties of the composites, and the relatively soft organic core often make contributions to the improvements of impact strength.

#### 3.1.2. Enhanced Tribological Properties

**Figure 8:**
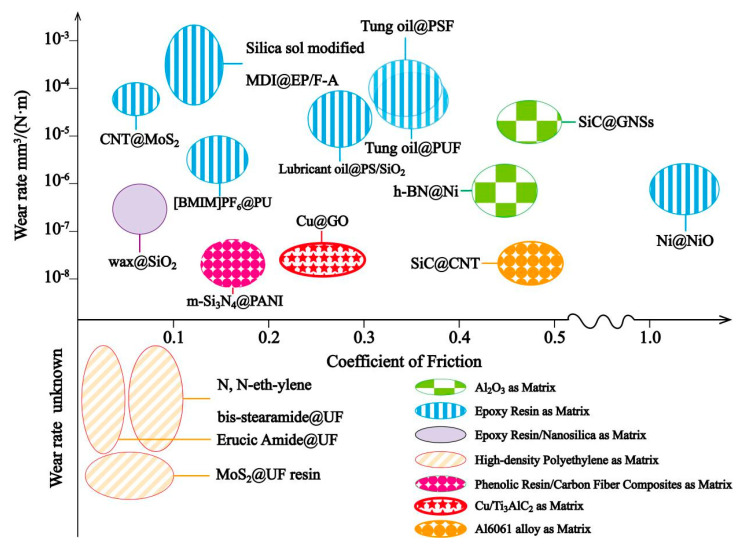
Tribological properties of different matrixes enhanced by core–shell particles.

Core–shell micro/nanoparticles with characteristic structural advantages are of great significance to improve the tribological properties of lubricating materials. In general, there are two ways for core–shell particles to improve the tribological properties of the matrix: in the form of micro/nanocapsules and core–shell particles with solid cores. The commonly used method is micro/nanocapsules [55,56,57,58,59,60,61]. The core–shell particles with solid cores to improve the tribological properties of solid matrix are less reported at present relatively. The parameters of some core–shell particles strengthening the tribological properties of the matrix reported so far are summarized in Table 2 and Figure 8 and described in detail.

**Figure 9:**
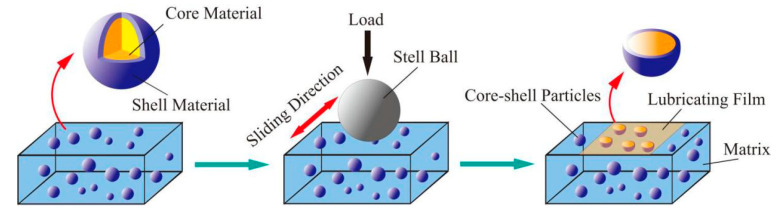
Formation of smooth agent film during sliding wear.

**Figure 10:**
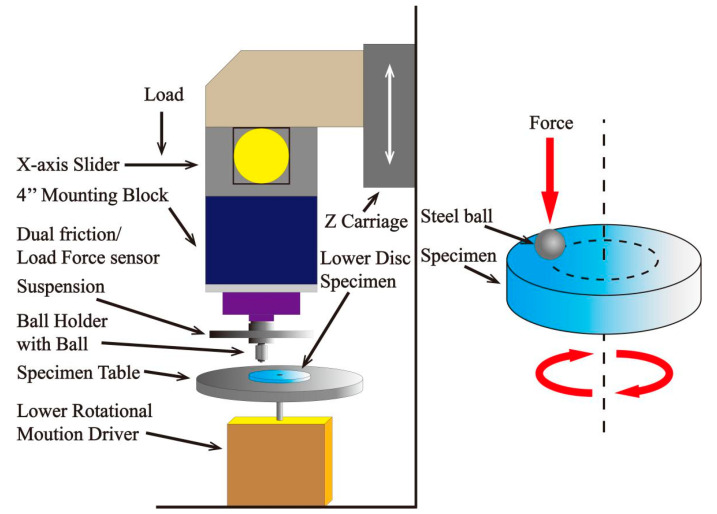
Schematic illustration of UMT3 tribometer for the ball-on-disc sliding test [61]. Reproduced with permission from [Abolhassan Imani et al.], [Composites Part A: Applied Science and Manufacturing]; published by [Elsevier], 2018.

The introduction of nano/microcapsules into a matrix can improve the friction properties of the composites because the lubricant oil encapsulated in the particles can be released with the broken off nano/microcapsules caused by the friction process as shown in Figure 9. What is more, the broken empty capsules can collect the debris from the friction pairs to reduce wear. For nano/microcapsules composites, the friction properties depend deeply on the type of lubricant oil coated inside and the dispersion properties of the nano/microcapsules in the matrix. LO@PU microcapsules were embedded in epoxy resin to obtain composites with self-lubricating and self-healing properties [55]. During the friction process, the released LO formed a transfer film on the friction pair to prevent direct contact between the sample and friction pair, resulting in the low COF of 0.07 (20 wt %). Different microcapsules were added to high-density polyethylene (HDPE) to form self-lubricating composites with an ultralow COF [56]. The results showed that erucic@UF (urea formaldehyde resin) microcapsules could reduce the COF of HDPE to 0.03. This could be attributed to the formation of a smooth layer between the friction pairs. Li et al. [57,58,59] conducted a series of studies on microcapsules/epoxy resin composites and greatly improved the lubricating properties of the composite materials because of the newly formed oil film between the epoxy and sliding surface. What is more, they found that the wear debris of shells could act as liquid additives in the oil to improve the wear resistance of the composites. 1-butyl-3-methylimidazolium hexafluorophosphate ([BMIM]PF_6_)@PU high-temperature-resistant microcapsules were prepared to greatly improve the fiction properties of epoxy resin (COF of 0.151, 72.7% lower than that of pure epoxy resin (EP)) [60]. Imani and his team [61] synthesized wax@SiO_2_ nanoparticles and embedded them in the EP/SiO_2_ matrix. The COF and wear rate of the composites were both reduced significantly due to the development of the thin and continuous transfer film on the steel ball and the good balance among basic mechanical properties. Their experimental setup was shown in Figure 10. Nano/microcapsules can improve the tribological properties of the matrix greatly due to their lubricant oil core materials. However, the introduction of capsules will greatly reduce the mechanical properties of the matrix because of the liquid core. Consequently, micro/nanoencapsulated lubricating composites are limited in their applications in specific working conditions that need high mechanical properties.

Different from nano/microcapsules, the solid core–shell particles can reduce the COF of the matrix while maintaining or improving the mechanical properties. Conventionally, MoS_2_ is an ideal solid lubrication addictive in the polymer matrix due to its superior self-lubricating ability [41]. Therefore, research studies have been conducted on the encapsulation of MoS_2_ into urea formaldehyde (UF) resin to improve the chemical stability of MoS_2_ in a humid environment and the friction properties of composites when MoS_2_@UF resin core–shell particles are used as fillers [62]. The results showed that the composites containing the MoS_2_@UF resin exhibited lower sliding friction and smoother surface morphology because of the protection provided by shells, which delayed the oxidation of the MoS_2_ crystals. MoS_2_ can also be used as a shell material; CNF@MoS_2_ core–shell particles were prepared via the one-step hydrothermal method to enhance the anti-friction and anti-wear properties of epoxy resin [63]. The MoS_2_ shell could provide the good interfacial adhesion between CNF@MoS_2_ particles and epoxy resin; what is more, the MoS_2_ debris desquamated during the sliding process could form a dense and uniform lubricating film to reduce the COF and wear rate. In addition to MoS_2_, epoxy resin reinforced with Ni@NiO magnetic nanoparticles was presented [54]. The mechanical and tribological properties of the composites were both improved greatly (the friction coefficient was reduced by 36.4%, the wear resistance increased by 22.2-fold, the hardness increased by 37.8%, and the elastic modulus increased by 16.3%), because the Ni@NiO particles filled up the cracks and defects produced during the friction process and formed a smooth transfer film, which improved the tribological properties of Ni@NiO/EP composites. Aiming at the contradiction between the tribological and mechanical properties of traditional self-lubricating ceramic composites, h-BN@Ni powders were fabricated by an electroless plating technique as fillers in the ceramic [64]. By adding h-BN@Ni powders, the microstructure of the ceramic composite was more homogeneous than that of the composite containing uncoated h-BN powders. In order to improve the poor thermal conductivity of the phenolic resin/carbon fiber (PF/CF) composites, m-Si_3_N_4_@PANI core–shell particles were synthesized [65]. The results indicated that the addition of m-Si_3_N_4_@PANI improved the thermal conductivity, electrical conductivity, COF, and wear rate of PF/CF composites effectively (thermal conductivity of 3.164 Wm^−1^·K^−1^, electrical conductivity of 5.33 × 10^−6^ S/m, COF of 0.1681 (reduced by 48.48%) and wear rate of 1.13 × 10^−8^ mm^3^/Nm (reduced by 68.08%)). A novel Cu matrix composite with low COF (0.2) was prepared by hot-press sintering the core–shell Cu@GO particles and Cu decorated Ti_3_AlC_2_ powders [66]. The GO and Ti_3_AlC_2_ could form continuous and compact transfer films on the wear surface, thus reducing the COF and wear rate. Zhang et al. [67] fabricated SiC@GNSs by a wet ball milling process as fillers in alumina matrix composites, and the composites exhibited superior tribological properties because of the combined effect of graphene nanosheets (GNSs)-rich tribofilms, which improved the microstructure and mechanical properties of the composites. Similarly, SiC was coated in carbon nanotubes (CNT) to obtain SiC@CNT nanoparticles using a CVD method and mixed with Al6061 alloy to fabricate SiC@CNT/Al6061 composites via spark plasma sintering [68]. The COF and wear rate of the composites decreased due to the transition of the wear mechanism from adhesive wear to abrasive wear because of the uniform dispersion of SiC@CNT debris between the sample and the friction pair.

In summary, the friction mechanism of core–shell nano/microparticles in a solid matrix is the formations of transfer films or lubricating layers between composites and friction pairs to separate the samples and friction pairs to avoid direct contact. However, for nano/microcapsules, the transfer films or lubricating layers are mainly generated by organic cores (lubricant oil) and shells, and both the matrix (most of them are EP and other polymer) and capsules cannot stand the high temperatures and loads. What is more, the wear resistance of the composites need further improvements. All those factors will limit the applications of nano/microcapsule self-lubricating materials in the mechanical devices that need to operate under high temperature or for a long time. As for core–shell solid particles, most of them are inorganic and can maintain properties under high temperatures; however, the tribological properties of the composites still need to be enhanced before practical applications. Consequently, it is necessary to further develop core–shell self-lubricating materials with high temperature and load resistances to meet the requirements of friction components in aerospace, transportation, and other fields in the future.

### 3.2. Additives in Liquid Lubricants

**Figure 11:**
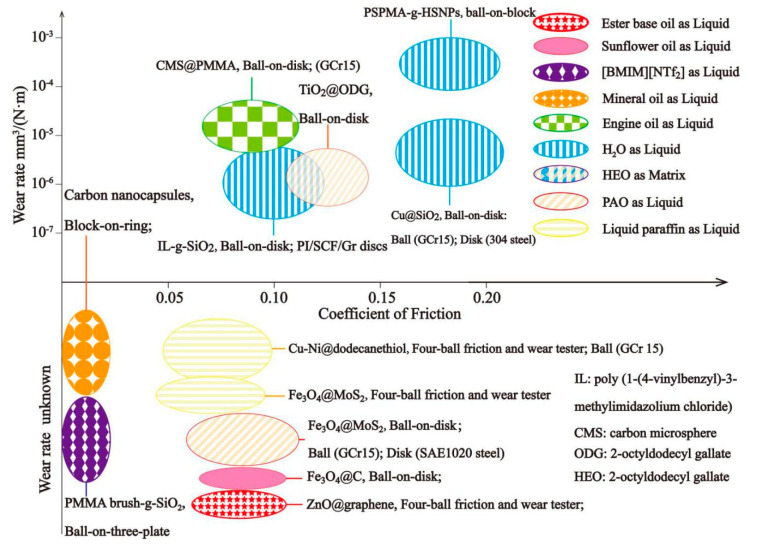
Tribological properties of different lubricating liquid enhanced by core–shell particles.

Liquid lubricants are of great importance for friction pairs because of their abilities to reduce COF, wear, contamination, and eliminate excess heat of the systems. However, suitable additives are needed to enhance the certain properties of liquid lubricants such as the wear resistance, oxidation resistance, and anti-corrosion [69]. Many additives such as carbon-based particles [70,71,72,73], metal composite nanoparticles [74,75], metal oxides [76,77], and MoS_2_ [78,79] have been studied during the last few decades. Nonetheless, in turn, the uneven distribution and aggregation of the additives will reduce the lubricating properties of liquid lubricants. In order to overcome these problems, core–shell nano/microparticles as lubricant additives are proposed. Herein, some of the core–shell nano/microparticles as additives in liquid lubricants studied by now are summarized in Table 3 and Figure 11 and discussed in detail.

**Figure 12:**
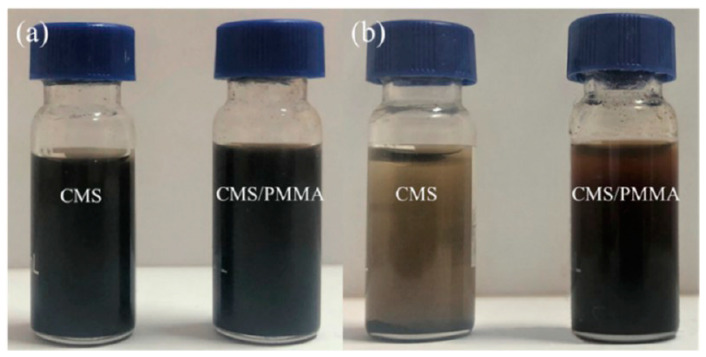
Digital pictures of carbon microspheres (CMS) and CMS/PMMA dispersions (**a**) immediately after sonication and (**b**) 8 weeks after sonication [80]. Reproduced with permission from [Yuefeng Gu et al.], [The Journal of Physical Chemistry C]; published by [American Chemical Society], 2019.

Nano/microparticles tend to reduce their surface free energy by aggregation in non-polar oil liquid, and the metal–organic tribofilm between the lubricant oil and friction pair could be easily worn off by the aggregated particles, thus reducing the friction properties of the lubricant oil. To solve the aggregation problem of nano/microparticles, many core–shell particles have been reported by researchers around the world. Gu et al. [80] coated carbon microspheres (CMS) with PMMA composites that had lipophilic groups such as aminopropyl (APTES) and−COOCH_3_ (PMMA), leading to the stable existence of carbon particles in engine oil, as shown in Figure 12. TiO_2_ particles were coated with 2-octyldodecyl gallate (ODG) and used as additives in different oil to not only improve the dispersion stabilities but also introduce the phenol-related tribochemical reactions [81]. The results showed that the alkyl groups on ODG@TiO_2_ nanoparticles slowed down the sedimentation kinetic, and the additional ODG layers on TiO_2_ nanoparticles facilitated the TiO_2_ nanoparticles to incorporate with the zinc dialkyldithiophosphate (ZDDP)-derived tribofilms. Bimetallic Cu–Ni nanoparticles surface-capped with dodecanethiol were presented to increase the load-carrying capacity and tribological properties of liquid paraffin, and the paraffin could maintain a COF of 0.07 under different loads (196 N, 392 N) [82]. The results showed that the Cu-Ni@dodecanethiol particles almost had no sign of aggregation and exhibited good dispersibility in different non-polar solvents. Hydrophobic Cu nanoparticles were incorporated into silica spheres to solve the problem that the di-n-hexadecyldithiophosphate-modified Cu could not be used as a water-based lubricant, and the large amount of Si–OH groups on the SiO_2_ surfaces could contribute to a significant improvement of dispersion of Cu@SiO_2_ particles in water-based lubricants [84]. Furthermore, the wear rate was reduced up to 94.1% when used as a lubricant additive in distilled water.

**Figure 13:**
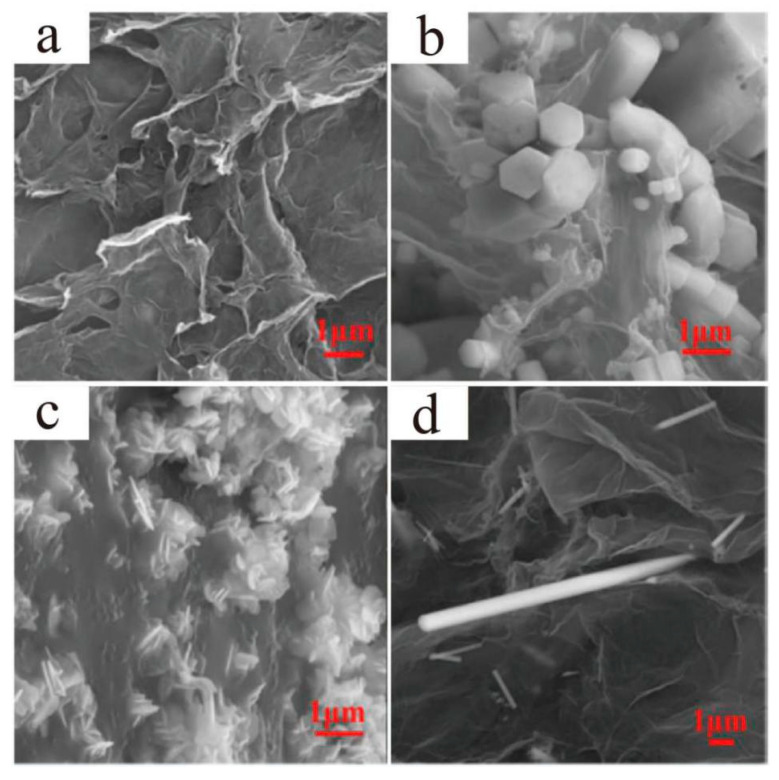
Micromorphology of synthesized nanoparticles: (**a**) graphene, (**b**) prism-like ZnO@graphene, (**c**) sheet-like ZnO@graphene, (**d**) rod-like ZnO@graphene [86]. Reproduced with permission from [Baijing Ren et al.], [Tribology International]; published by [Elsevier], 2020.

To improve the dispersion property of the CNC in the mineral oil, the CNC particles were surface modified with alkyl chains and wet–mechanical ground to reduce the surface energy [85]. It was proved by researchers that sheet-like ZnO@graphene nanoparticles were more suitable for dynamic friction environments in ester base oil while local agglomeration could be seen in the prism-like nanoparticles, as can be seen in Figure 13 [86], which meant that the morphologies of the particles could also affect the dispersion properties.

**Figure 14:**
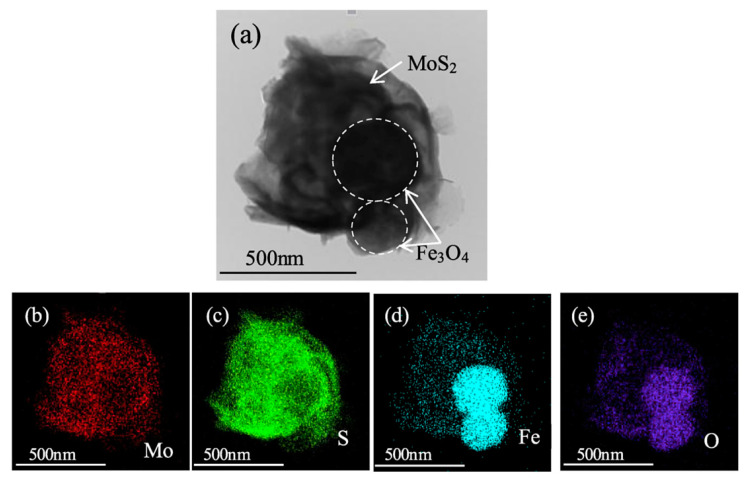
TEM (**a**) image and corresponding elemental mapping (**b**–**e**) images of Fe_3_O_4_@MoS_2_ nanocomposites [87]. Reproduced with permission from [Yufu Xu et al.], [Tribology International]; published by [Elsevier], 2018.

Generally, the Fe_3_O_4_ nanoparticles are hard to disperse and maintain stable in the liquid lubricants because of the magnetic properties and high surface energy, which will affect the tribological properties of Fe_3_O_4_ nanoparticles when used as lubricant additives. To overcome the problems, Fe_3_O_4_ was encapsulated into MoS_2_, the core–shell particles were used as additives for different liquid lubricants (PAO_4_ (Figure 14) and liquid paraffin) [87,88], and both of them showed great dispersion and tribological properties. In addition to MoS_2_, Fe_3_O_4_ particles were also coated with carbon layers via hydrothermal carbonization to enhance the dispersion property in sunflower oil [83]. What is more, the synergistic effect between the carbon layer and magnetic Fe_3_O_4_ could also improve the tribological performance of Fe_3_O_4_@C particles.

**Figure 15:**
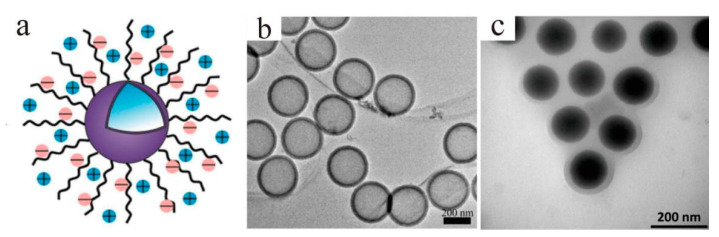
(**a**) Schematic and (**b**) TEM representation of PSPMA-g-HSNPs [89]. Reproduced with permission from [Guoqiang Liu et al.], [The Journal of Physical Chemistry B]; published by [American Chemical Society], 2014. (**c**) TEM image of PMMA brush-g-SiO_2_ [91]. Reproduced with permission from [Amir Beheshti et al.], [Tribology International]; published by [Elsevier], 2020.

Surface modification is another effective means to prevent aggregation, as it can create a buffer around particles and make their external polarities the same. Core/shell charged polymer brushes-grafted hollow silica nanoparticles (PSPMA-g-HSNPs) (Figure 15a,b) were used as lubricant additives in distilled water and reduced both the COF and wear rate by about 50% under a high load (1.66 GPa) [89]. Poly(1-(4-vinylbenzyl)-3-methylimidazolium chloride) grafted mesoporous SiO_2_ particles were used as lubricant additives in distilled water, and the results proved that the grafted layers facilitated the formation of a tribofilm, which separated the direct contact and retarded the oxidation of the friction pairs [90]. To obtain the ultralow COF of the lubricant, Beheshit and co-workers [91] fabricated PMMA brush-grafted SiO_2_ particles (Figure 15c) as additives in [BMIM][NTf_2_], and the COF was only 0.01, which was almost reduced by 85.7%.

Generally, liquid lubricants with lubricating additives can achieve very low COF even under 0.01. However, traditional liquid lubricant additives tend to aggregate during the friction process, and the main aim of core–shell particles is to prevent the aggregation by surface modification or coating the core materials with shells containing lipophilic/hydrophilic groups. What is more, compared to the solid self-lubricating materials, additives in liquid lubricants require continual supplements, during which process the amount of additives needed to be replenished is hard to control. When the concentration of additives is too low, the tribofilms are hard to form, leading to the direct contact of friction pairs. Correspondingly, when the concentration is too high, nanoparticles tend to aggregate, which will lead to the sharp increase of COF and wear rate. Therefore, it cannot be ignored when the core–shell nano/microparticles come into use as lubricant additives.

### 3.3. Composite Abrasives in Chemical Mechanical Polishing

**Figure 16:**
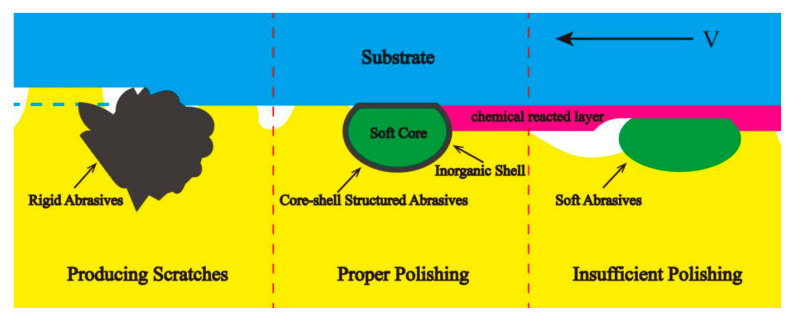
Probable material removal mechanism of the chemical mechanical polishing (CMP) process [92]. Reproduced with permission from [Hyunseop Lee et al.], [International Journal of Precision Engineering and Manufacturing]; published by [Springer Nature], 2016.

Chemical mechanical polishing (CMP) uses chemical etching and mechanical forces to smooth silicon wafers or other substrate materials during processing. The chemicals in the polishing fluid react with the surface of the substrate to form substances that are easier to remove, while mechanical and physical friction occur between the abrasive particles in the polishing fluid and the substances generated by the chemical reactions, as shown in Figure 16. The standards for judging the quality of polishing are the root mean square (RMS) surface roughness and material removal rate (MRR).

The requirements for CMP abrasives are strict:

1. The abrasives must be neither harder than the substrate to cause severe mechanical damage nor too soft to remove the chemically generated surface.
2. The abrasives do not react with the composition of the polishing fluid.
3. The abrasives must be well dispersed in the polishing fluid and not agglomerate.
4. The abrasives do not dissolve in the polishing fluid.

**Figure 17:**
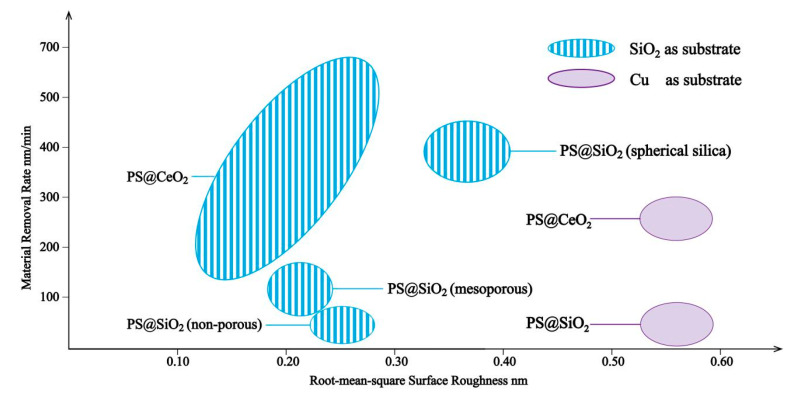
CMP behavior of the core–shell particles as abrasives.

Compared with conventional abrasives, core–shell structured abrasives have many advantages. The elastic moduli of the abrasives can be adjusted by controlling the hardness of core materials and the thickness of the shells to fit the substrates [93]. The soft core can deform elastically and increase the real contact area between abrasives and substrates to reduce the contact stress and prevent the appearance of the scratches on substrates. The existence of the shell materials can also improve the dispersion of abrasives and prevent the physical dissolving and chemical reaction between abrasives and polishing fluid. What is more, the relatively rigid shell ensures that the chemical reacted layers can be totally removed. Compared to spherical core–shell structured abrasives, the traditional abrasives such as SiO_2_ and CeO_2_ have irregular shapes and are rigid, which will easily scratch the substrate during the polishing process. The focuses of the study are mainly for non-rigid polymer core/inorganic shell materials to gain proper hardness for polishing. Table 4 and Figure 17 show the parameters of the core–shell composite abrasives in chemical mechanical polishing.

**Figure 18:**
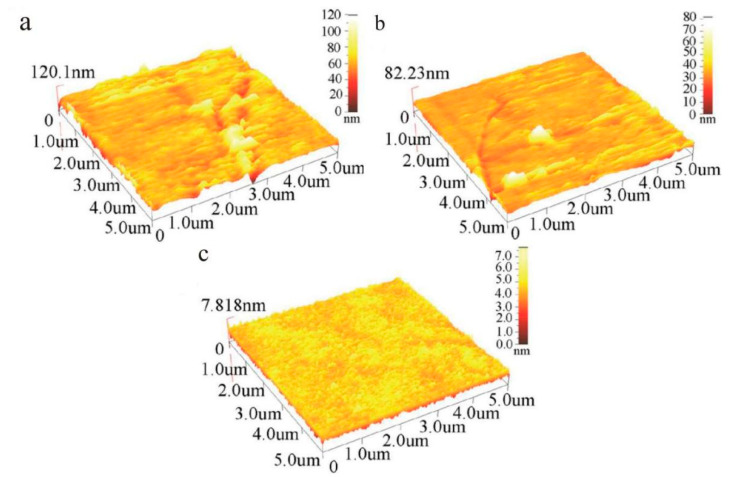
Atomic force microscope (AFM) images of blanket copper wafers: (**a**) before CMP, root mean square (RMS) roughness 8.47 nm, (**b**) after CMP by colloidal silica abrasive, RMS roughness 4.27 nm, (**c**) after CMP by PS@SiO_2_ abrasive, RMS roughness 0.56 nm [16]. Reproduced with permission from [Lei Zhang et al.], [Applied Surface Science]; published by [Elsevier], 2011.

The current structure of core–shell abrasives in chemical mechanical polishing is mainly an organic core/inorganic shell. Chen et al. [95] studied the CMP performance of PS@SiO_2_ abrasives; in their research studies, two different SiO_2_ as shell materials were used. They found that the abrasives with mesoporous silica shells and non-porous silica shells had similar RMS, while the MRR varied widely, which could be attributed to the mesoporous silica’s adsorption of active chemical constituents in slurry, improving the chemical reactivity in the real contact area, and the enhanced chemical reaction improved the material removal process. In addition to an enhanced chemical reaction, the morphologic differences of a silica shell can also affect the CMP performance of abrasives. Core–shell structured PS@SiO_2_ particles with spherical silica as the shell were synthesized [94] and achieved a higher MRR of 387 ± 44 nm/min, which might be attributed to the enhanced mechanical action in CMP caused by the rough appearances of the spherical silica shell. The CMP performance of PS@SiO_2_ on the Cu substrate was also investigated [16] and achieved a great reduction of RMS surface roughness (from 4.27 to 0.56 nm) by 86.89% compared with pure SiO_2_ abrasives (Figure 18).

Another commonly used shell material is CeO_2_; Chen et al. [96,98] presented PS@CeO_2_ abrasives and applied them to different substrates (SiO_2_ and Cu), and both of them possessed great properties during the CMP process. Other PS@CeO_2_ particles were fabricated and used as abrasives for the CMP process of a SiO_2_ substrate [97], and it had a great improvement on MRR (440.3% higher than pure CeO_2_), which could be attributed to an increased filling factor with more CeO_2_ particles interacting with the surface.

In addition to PS, PMMA can be chosen as a core material as well. Armini and her team [99] studied PMMA@SiO_2_ and examined the dependence of CMP performance on both polymer cores and silica shells. They found that the elastic moduli of the core/shell organic/inorganic composites were much closer to those of the polymer cores and lower than shell materials, while the inorganic shells stiffened the cores.

Generally, the CMP is mainly affected by the rigidity of abrasives and particle surface activity [100]. The core–shell nano/microparticles are alternative materials for CMP because of their adjustable structures and mechanical/chemical properties. The synergistic effect of the core and shell can greatly improve the CMP performance because the elastic cores can increase the real contact area, while inorganic shells can stiffen the mechanical properties of the abrasives. The design idea of the core–shell CMP abrasives are to find the balance between soft cores and rigid shells. Currently, most of the shell materials are traditional CMP abrasives such as SiO_2_ and CeO_2_, and the cores are PS; consequently, this area is still well worth expanding to find new core and shell materials.

### 3.4. Basic Units in Bulk Composites

**Figure 19:**
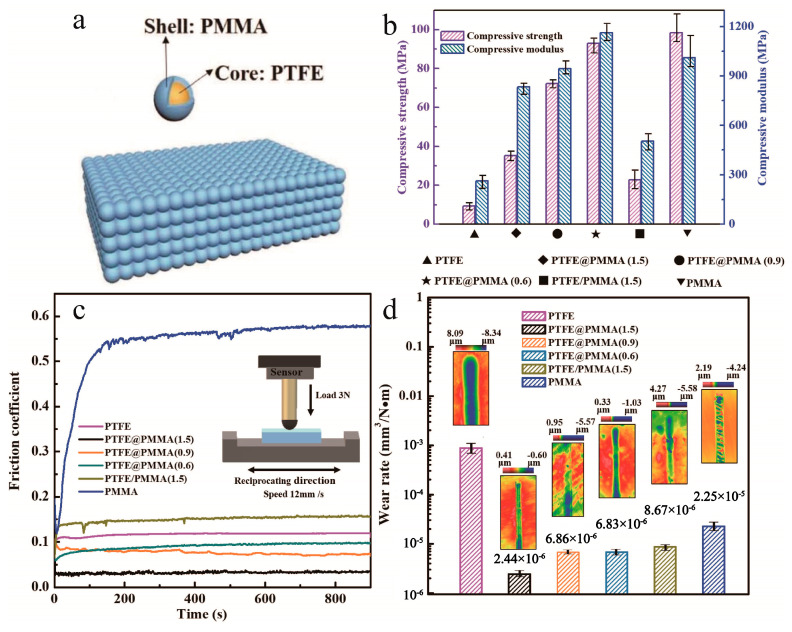
(**a**) Microstructural diagram of the PTFE@PMMA composite, (**b**) compression curves of the specimens, (**c**) variations in the friction coefficients of the test specimens over time, (**d**) white light interferometry images and wear rates of the specimens [105]. Reproduced with permission from [Lin Zhang et al.], [Nanoscale]; published by [Royal Society of Chemistry], 2019.

Core–shell micro/nanoparticles are widely used as fillers in a solid matrix to obtain self-lubricating properties and high mechanical performances. However, in most of the cases, it is hard to achieve both goals at the same time [101,102,103,104]. To solve this problem, novel PTFE@PMMA core–shell mircoparticles composite materials were synthesized by Zhang et al. [105] (shown in Figure 19). Instead of applying the particles as fillers in the matrix, they fabricated the bulk composite using PTFE@PMMA as basic units by compression molding. The composite possessed an ultralow COF of 0.03 and superior mechanical properties (compressive strength increased from 9 to 90 MPa compared with pure PTFE). PTFE@polyacrylate particles were also presented as basic units in composite [106] and improved the thermal stability and tribological properties obviously (friction coefficient dropped from 1.33 to 0.39 compared with pure polyacrylate).

Similar to the additives in solid matrix, the bulk composites with core–shell nano/microparticles as basic units reduce the COF by forming the layers of transfer lubricating films and prevent the rubbing of surfaces from direct contact. Although the research studies of core–shell structured nano/microparticles as basic units in bulk composites are rarely reported at present, it is a very promising way to improve the properties of composite materials and can be further studied in the future.

## 4. Conclusions

The goal of this review is to emphasize the synthesis methods and the tribological applications of core–shell micro/nanoparticles in recent years. Given that the core–shell structured particles have the unique properties of morphology, adjustable structure, and material storage capacity, the study on applications of core–shell micro/nanoparticles in tribology will eventually bring great breakthroughs in the future.

The synthesis of core–shell particles can be summarized in two steps: the core material is synthesized, and then the shell is synthesized on the basis of the core material, or a shell layer is deposited over a pre-formed core. The specific synthesis methods can be divided into four categories according to different principles: solid phase reaction, liquid phased reaction, gas phase reaction, and mechanical mixing.

As for the applications, the current applications of core–shell micro/nanoparticles mainly focus on the following aspects: filler in a solid lubricant matrix, additive in liquid lubricants, composite abrasive in chemical mechanical polishing, and basic units in bulk composite. When used as fillers in a solid matrix, core–shell micro/nanoparticles can enhance the mechanical and tribological properties of solid materials due to the structure and good dispersion properties of the particles. Compared with ordinary particles, core–shell micro/nanoparticles possess the shell materials that can interlock the matrix and particles as well as improve the dispersion of particles in the matrix, resulting in the improvement of both the mechanical and tribological properties of solid materials. The focus of core–shell micro/nanoparticles application as an additive in liquid lubricants is mainly on the improvement of particle dispersion in liquid. Generally, liquid lubricants with core–shell structured lubricating additives can achieve very low COFs (0.01). However, additives in liquid lubricants require continual supplementation, and during this process, the amount of additives needed to be replenished is hard to control. Compared with traditional abrasives in a chemical mechanical polishing process, core–shell particles have many advantages: adjustable elastic moduli by controlling the relatively soft core materials and the thickness of the shells and great dispersion due to the shell materials. The existence of shell materials can also prevent the chemical reaction between abrasives and polishing fluid and physical dissolving. The spherical shape of core–shell structured abrasives can act as ball bearings to reduce the mechanical damages. These all give a promising application future of core–shell structured abrasives in chemical mechanical polishing. Acting as basic units in bulk composite is a newly proposed application of core–shell micro/nanoparticles in the tribological field, which is a very novel way to simultaneously achieve remarkable mechanical properties and outstanding lubrication properties, and this application deserves more attention from researchers in the future.

In general, the tribological application of core–shell micro/nanoparticles is one of the promising methods to improve the lubrication and mechanical properties of the materials due to their relatively simple synthesis method, diverse composition, adjustable performance, and synergistic effect among components. With the advances that the research works have made, it is expected that the theoretical studies will eventually benefit from commercial applications and research studies of core–shell micro/nanoparticles in the future. It is hoped that this review can render its trifling service of helping development in this important field.

## Figures and Tables

**Table 1:** Parameters of enhanced mechanical properties.

Composites		Mechanical Properties (Compared with Pure Matrix)	Ref.
Filler (Content)	Matrix		
TiO_2_@DPP (0.10 wt %)	PC	Elongation at break: 126.4% (27.7 higher) Tensile strength: 78.0 MPa (14.7% higher)	[43]
SiC@SiO_2_-NH_2_ (10 wt %)	PI	Tensile strength: 185 MPa (77.1% higher)	[44]
SiO_2_@PMMA (5 wt %)	PVC	Elongation at break: 36.8% (105.6% higher) Tensile strength: 42.9 MPa (100.5% higher)	[45]
SiO_2_@PDMAEMA (2 wt %)	PAA	Compressive strength: 349.96 kPa (113.5% higher);	[46]
CaCO_3_@PMMA (0.75 wt %)	PP	Elongation at break: 675% (68.8% higher)	[47]
CaCO_3_@PMMA (1 wt %)	PP	Tensile strength: 55 MPa (71.9% higher)	[47]
CaCO_3_@PS (0.25 wt %)	PS	Elongation at break: around 50% (11% hgiher)	[48]
CaCO_3_@PS (1 wt %)	PS	Tensile strength: around 35 MPa (34% higher)	[48]
TiO_2_@PS (1 phr)	LLDPE/PLA	Elongation at break: 980% (95.2% higher) Tensile strength: 16.5 MPa (94.1% higher)	[49]
Starch@PMA (20 wt %)	PPC	Young’ s modulus: 529 MPa (50.4-fold higher) Tensile strength: 21.5 MPa (3.2-fold higher) Toughness: 82 J·m^−3^ (44% higher)	[50]
NR@PMMA-co-PMPS (5 wt %)	PMMA	Impact strength: 5.40 kJ/m^2^ (17.9% higher) Elongation at break: 6.25% (91% higher) Tensile strength: 54.59 MPa (2% higher)	[51]
BAR@MMA (3 wt %)	CDA	Impact strength: around 145 J/m (115% higher)	[52]
Poly(n-butyl acrylate)@PMMA (16.66 wt %)	PBT	Impact strength: 75.20 kJ/m^2^ (8.64-fold higher)	[53]
Ni@NiO (5 wt %)	EP	Hardness: 0.5 GPa (37.8% higher) Elastic modulus: 5.6 GPa (16.3% higher) Wear resistance: increased by 22.2-fold	[54]

DPP: diphenylphosphinic; PC: polycarbonate; PI: polyimide; PMMA: polymethyl methacrylate; PVC: polyvinyl chloride; PDMAEMA: poly(2-(dimethylamino)ethyl methacrylate); PAA: polyacrylic acid; PP: polypropylene; PS: polystyrene; LLDPE: linear low-density polyethylene; PLA: poly lactic acid; PMA: poly-(methyl acrylate); EP: epoxy resin; PPC: poly(propylene carbonate); NR: natural rubber; BAR: butyl acrylate rubber; CDA: cellulose diacetate; PBT: polybutylece terephthalate; PMMA-co-PMPS: poly(methyl methacrylate)-co-poly(3-trimethoxy silylpropyl methacrylate).

**Table 2:** Parameters of enhanced tribological properties.

Composites		Friction Condition		Friction Properties (Compared with Pure Matrix)		Ref.
Filler (Content)	Matrix	Load	Sliding Speed	COF	Wear Rate mm^3^/(N·m)	
LO@PU (20 wt %)	EP	3 N	0.05 m/s	0.07 (90.65% lower)		[55]
N, N-ethylene bis-stearamide@UF (10 wt %)	HDPE	1 MPa	50 rpm	0.075 (25.4% lower)	·	[56]
Erucic Amide@UF (10 wt %)	HDPE	1 Mpa	150 rpm	0.03 (66.7% lower)	·	[56]
Tung oil@PSF (10 wt %)	EP	1.0 MPa	0.51 m/s	0.35 (23.9% lower)	10^−4^ (66.1% lower)	[57]
Tung oil@UF (10 wt %)	EP	1.0 MPa	0.51 m/s	0.38 (17.3% lower)	8.26 × 10^−5^ (78.6% lower)	[58]
Lubricant oil@PS/SiO_2_ (10 wt %)	EP	1.0 MPa	0.51 m/s	0.271 (52.5% lower)	2.73 × 10^−5^ (92.9% lower)	[59]
[BMIM]PF_6_@PU (30 wt %)	EP	1.0 MPa	0.76 m/s	0.151 (72.7% lower)	4.81 × 10^−6^ (99.4% lower)	[60]
wax@SiO_2_ (10 wt %)	EP/SiO_2_	4 N	0.12 m/s	0.074 (87.7% lower)	4.39 × 10^−7^ (three orders of magnitude lower)	[61]
MoS_2_@UF resin (10 wt %)	HDPE	1.05 MPa	150 rpm	0.01 (89.5% lower)	·	[62]
CNF@MoS_2_	EP	4 N	200 rpm	0.075 (82.1% lower)	8.6 × 10^−5^ (87.5% lower)	[63]
Ni@NiO (5 wt %)	EP	1.0 MPa	1.0 m/s	1.05 (36.4% lower)	10^−6^	[54]
h-BN@Ni (5 vol %)	Al_2_O_3_/C	20 N	200 rpm	0.45 (6.25% lower)	10^−6^ (26.2% lower)	[64]
m-Si_3_N_4_@PANI (2.0 wt %)	Phenolic resin	·	·	0.1681 (48.48% lower)	1.13 × 10^−8^ (68.1% lower)	[65]
Cu@GO (0.8 wt %)	Cu/Ti_3_AlC_2_	4 N	200 rpm	0.2 (about 50% lower)	2.0 × 10^−8^	[66]
SiC@GNSs (5 vol %)	Al_2_O_3_	90 N	0.1 m/s	0.45 (29.4% lower)	2.6 × 10^−5^ (90.1% lower)	[67]
SiC@CNT (5 vol %)	Al6061 alloy	98.1 N	200 rpm	0.45 (31% lower)	3.25 × 10^−8^ (45% lower)	[68]

LO: linseed oil; UF: urea formaldehyde resin; HDPE: high-density polyethylene; PSF: polysulfone; PS: polystyrene; PU: polyurethane; PANI: polyaniline; EP: epoxy resin; m-Si_3_N_4_: (3-aminopropyl) triethoxysilane-modified β-Si_3_N_4_; CNF: carbon nanofiber; GO: graphene oxide; GNSs: graphene nanosheets; CNT: carbon nanotubes.

**Table 3:** Parameters of additives in liquid lubricants.

Lubricant		Friction Condition		Friction Properties (Compared with Pure Liquid)		Ref.
Additive (Content)	Liquid	Load	Sliding Speed	COF	Wear Rate mm^3^/(N·m)	
C@PMMA (0.2 wt %)	Engine oil	30 N	0.08 m/s	0.112 (21.7% lower)	1.57 × 10^−5^ (41.42% lower)	[80]
TiO_2_@ODG (1 wt %)	PAO/HEO	100 N	0.05 m/s	0.12 (18.5% lower) /0.10 (7.4% lower)	3.44 × 10^−6^ (17.0% higher) /8.3 × 10^−7^(2.1% lower)	[81]
Cu-Ni@dodecanethiol (0.1 wt %)	LP	196 N/392 N	1450 rpm	0.07 (61.1% lower) /0.07(39.1% lower)	·	[82]
Fe_3_O_4_@C (0.25 wt %)	Sunflower oil	5 N	300 rpm	0.077 (15.4% lower)	·	[83]
Cu@SiO_2_ (0.4 wt %)	H_2_O	4 N	0.02 m/s	0.17 (56.4% lower)	·	[84]
CNC (0.05 wt %)	Mineral oil	261 MPa	1.65 m/s	0.01 (25.9% lower)	·	[85]
ZnO@graphene (0.5~1.0 wt %)	Ester base oil	392 N	1200 rpm	0.075 (25% lower)	·	[86]
Fe_3_O_4_@MoS_2_ (1 wt %)	PAO_4_	20 N	0.05 m/s	0.08 (56.5% lower)	·	[87]
Fe_3_O_4_@MoS_2_ (0.08 wt %)	LP	·	·	0.066 (30.3% lower)	·	[88]
PSPMA-g-HSNPs (0.5 wt %)	H_2_O	1.66 GPa	0.05 m/s	0.173 (49.9% lower)	8.02 × 10^−4^ (52.7% lower)	[89]
IL-g-SiO_2_ (2 wt %)	H_2_O	90 Mpa	0.15 m/s	0.10 (41.2% lower)	2.7 × 10^−6^ (28.8% lower)	[90]
SiO_2_@PMMA (0.3 wt %)	[BMIM][NTf_2_]	2 N	0.05 m/s	0.01 (85.7% lower)	·	[91]

PMMA: polymethyl methacrylate; ODG: 2-octyldodecyl gallate; LP: liquid paraffin; IL: poly (1-(4-vinylbenzyl)-3-methylimidazolium chloride); PSPMA-g-HSNPs: poly(3-sulfopropyl methacrylate potassium salt) brushes-grafted hollow silica nanoparticles; CNC: carbon nanocapsules.

**Table 4:** Parameters of composite abrasives in chemical mechanical polishing.

Core–Shell Particles	Substrate	RMS Surface Roughness (nm)	MRR (nm/min)	Ref.
PS@SiO_2_ (spherical silica)	SiO_2_	0.37 ± 0.03 (63% lower than PS; 22.92% lower than SiO_2_)	387 ± 44	[94]
PS@SiO_2_	Cu	0.56 (86.89% lower than SiO_2_)	45	[16]
PS@SiO_2_ (mesoporous)	SiO_2_	0.22 ± 0.02 (42.11% lower than SiO_2_)	123 ± 15 (68% higher than SiO_2_)	[95]
PS@SiO_2_ (non-porous)	SiO_2_	0.25 ± 0.03 (34.21% lower than SiO_2_)	47 ± 13 (36% lower than SiO_2_)	[95]
PS@CeO_2_	SiO_2_	0.15 ± 0.02 (40% lower than CeO_2_)	189 ± 19 (49% higher than CeO_2_)	[96]
PS@CeO_2_	SiO_2_	0.239 (60.6% lower than CeO_2_)	517.6 (440.3% higher than CeO_2_)	[97]
PS@CeO_2_	Cu	0.56 (70.37% lower than CeO_2_)	254	[98]

RMS: root mean square; MRR: material removal rate.
